# Measuring External Rotation of the Fibula and Fibular Length in Bilateral Computed Tomography Scans: How Reliable Is This Method?

**DOI:** 10.1097/BOT.0000000000002774

**Published:** 2024-02-02

**Authors:** Diederick Penning, Juul Molendijk, Jens A. Halm, Tim Schepers

**Affiliations:** Trauma Unit, Department of Surgery, Amsterdam UMC location Meibergdreef, Amsterdam, the Netherlands.

**Keywords:** fibular rotation, Syndesmosis, CT scan measurements, normal values, fibular length

## Abstract

**OBJECTIVES:**

During ankle fracture surgery, goals include accurate reduction and fixation of the fibula regarding rotation and fibular length. Bilateral postoperative computed tomography (CT) can be performed to assess fibular rotation using the talar dome angle, and fibular length. The aim of this study was to compare side-to-side differences of the fibular rotation and fibular length using bilateral CT scans of uninjured ankles.

**METHODS:**

**Design:**

Retrospective.

**Setting:**

Single center, Level I Academic Trauma Center.

**Patient Selection Criteria:**

Patients with bilateral CT scans of uninjured ankles.

**Outcome Measures and Comparisons:**

External rotation using the Nault talar dome method and fibular length using the coronal method of Prior et al. The average, difference, and ratio (injured side/healthy side) and interobserver variability were calculated.

**RESULTS:**

There were 83 patients included (166 ankles, mean age 47 years, 77.1% male). A random set of 66 ankles (33 CT scans) were used to measure interobserver variability. The mean degrees of external rotation ranged from 6.6 to 7.7, mean difference ranged from 1.4 to 3.4 degrees, mean ratio ranged from 1.1 to 1.5, and interobserver variability ranged from 0.27 to 0.65. For fibular length, the mean ranged from 24.6 to 25.8 mm, mean difference in fibular length ranged from 0.5 to 2.1 mm, mean ratio ranged from 1.0 to 1.1 mm, and interobserver variability ranged from 0.45 to 0.73.

**CONCLUSIONS:**

Using bilateral ankle CT scans, mean differences in fibular rotation using the Nault talar dome method were 1.4–3.4 degrees. The distal fibular length had a mean difference between both sides of 0.5–2.1 mm. Although the intraclass correlation's were low, the interleg differences between patients were small, making them useful for clinical practice.

**LEVEL OF EVIDENCE:**

Diagnostic Level III. See Instructions for Authors for a complete description of levels of evidence.

## INTRODUCTION

In patients with ankle fractures, to restore the native anatomical relationships of the fibula and tibia and regain physiological motion (and stability) of the syndesmosis, preventing the development of osteoarthritis and improving functional outcome, accurate reduction and fixation of the tibiofibular joint is necessary.^1,2^ In addition, an anatomical reduction and fixation of the fibula regarding length and rotation is paramount.^3,4^ Following open reduction and internal fixation, a percentage of ankle fractures will result in malreduction, which can lead to impaired function.^5–10^ Intraoperatively, great care should be taken to anatomically align the ankle in terms of osseous and ligamentous structures.^11,12^ Marmot et al showed that up to 30 degrees of rotation could go unnoticed per-operatively when using standard fluoroscopy.^13^ A postoperative computed tomography (CT) scan is advised in cases of doubt regarding the quality of reduction.^14,15^

Measuring the accuracy of tibiofibular reduction has been challenging, but bilateral postoperative CT imaging is a commonly used method.^16–18^ The use of CT is more sensitive compared with the use of conventional radiographs.^19^ When analyzing CT images, there are multiple methods to assess syndesmotic reduction, including the talar dome angle as described by Nault et al^15,20–27^ In addition, not only can incorrect reduction of the fibula in the incisura lead to rotational deformities, an incorrect rotational reduction of the fibula fracture leads to a rotational deformity. Correct fibular length is important to prevent a valgus malreduction of the ankle joint, talar tilt and shift, a widened medial clear space of the ankle, and subsequent posttraumatic osteoarthritis. The length of the distal fibula has often been measured using conventional radiographs, but can be measured with multiple methods including CT.^24,28^ Although the length of the distal fibula has been described,^24,29^ the (normal)side-to-side difference in length of the fibula of uninjured ankles remains unclear. This is important to study because the uninjured ankle could be used as a reference for reduction and for length in reconstructive surgery following a shortened fibula.

After the assessment of multiple methods to measure the rotation in the tibiofibular joint, the Nault talar dome angle has proven to be the most reliable.^15^ Therefore, the aim of this study was to compare (normal) side-to-side differences of the fibular rotation in bilateral CT scans of uninjured ankles. In addition, the length of the fibula in bilateral CT scans of uninjured ankles will be measured with the aim to define difference between both sides.

## MATERIALS AND METHODS

In this retrospective cohort study, patients were selected in a large Level 1 academic center. Patients with non–weight-bearing bilateral CT scan of the ankle for other indications than distal tibia or distal fibula fracture (eg, calcaneal fracture, forefoot injury, and midfoot injury) were included.

### Measurements

The measurements were performed in Agfa Health Care Xero viewer, on both ankles. Standardized reconstructions in 3 anatomical planes were created to ensure measurements at the correct level and/or angle. Before the start of the study, a meeting was scheduled to ensure that the measurements were made in a similar fashion by different observers.

**FIGURE 1.**
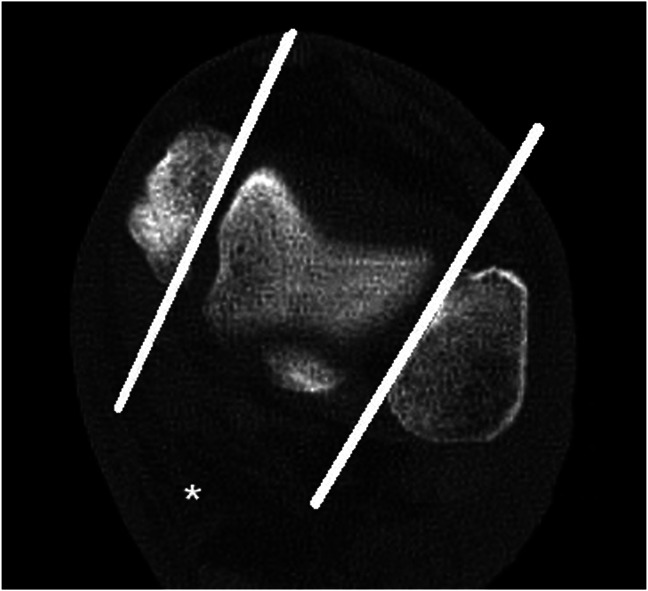
Nault talar dome measurement of rotation. The white lines represent the lines to measure the angle (*) between the talar (lateral) side of the medial malleolus and joint surface of the lateral malleolus at the level of the talar dome.

Because bilateral CT scans were used, sides were marked as “fracture side” or “healthy side,” though there were no (traumatic) abnormalities at the level of the ankle joint. The external rotation measurement was performed using the Nault talar dome method (Fig. 1).^21,23^ The axial view was used to measure the angle between the talar (lateral) side of the medial malleolus and joint surface of the lateral malleolus at the level of the talar dome. This angle was measured 5mm distal to the tibial plafond, which is comparable with the method used by Nault, where the level of the talar dome was used.^21^

**FIGURE 2.**
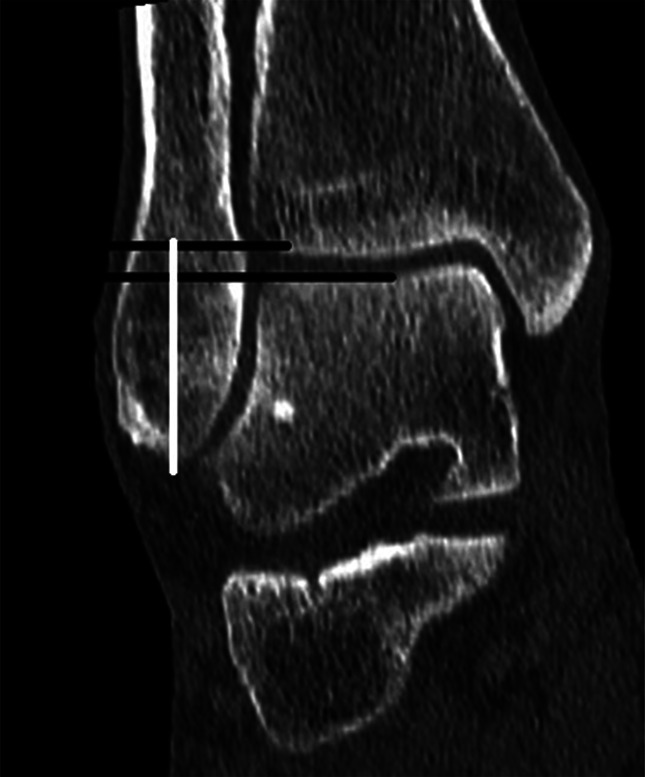
Measurement of the fibular length. The white line represents the fibular length, measured perpendicular to the tibial plafond.

The method used to measure the length of the fibula resembled the coronal method by Prior et al^26^. This method measured the length of the fibula from the tibial plafond to the distal tip of the fibula (Fig. 2).^24,26^ This method included drawing 2 lines; first, 1 horizontal line was drawn at the tibia plafond on the coronal or anteroposterior view, which resembled the proximal end of the fibular length, and second, a perpendicular line was drawn from the tip of the fibula to the horizontal tibia joint line, resembling the length of the distal fibula. The length in the anteroposterior plane where the Shenton line was visible and the “Weber's nose” were most prominent.^30^

After the measurements, both difference in mm and ratio (fibular length fracture side/fibular length healthy side) were calculated. Regarding the rotation and the fibula length, both the average, difference, and ratio (injured side/healthy side) were calculated per observer (degrees).

To assess interobserver variability, 4 independent reviewers performed the measurements. Investigator 1: fifth year medical student. Investigator 2: Second-year orthopaedic trauma resident. Investigators 3 and 4: Experienced orthopaedic trauma surgeons, both specialized in lower extremity fractures. Observers 1 and 2 performed measurements on the entire database. For the interobserver variability, a minimal sample of 30% was used, and therefore, a random set of 33 CT scans (66 ankles) was used. The interobserver variability was tested and expressed using the intraclass correlation (ICC) with 95% confidence interval (CI). The ICC was measured for the group of 4 investigators and between the separate observers. The following ICC interpretation was used: <0.5 poor, 0.5–0.75 moderate, 0.75–0.9 good, and >0.9 excellent.^31^

### Statistical Analysis

Statistical analysis was performed using IBM SPSS Statistics 28.0.0.1. Normality was assessed using the Kolmogorov–Smirnov test. To maintain uniformity, the mean and SD are displayed whenever most variables are normally distributed. The interobserver variability was also tested using SPSS. Therefore, the ICC with consistency was used in a 2-way mixed model. Statistical significance was defined as *P* < 0.05.

## RESULTS

### Patients

There were 83 bilateral CT scans retrieved. Therefore, a total of 166 ankles were measured. The mean age of these patients was 47 years (SD 13.5), and 77.1% were male patients.

### Side-to-side Comparisons

Table 1 summarizes the mean external rotation and mean fibular length per observer, including SD and range. The mean external rotation ranged from 6.6 to 7.7 degrees between the 4 observers (range 0.1–22.0 degrees and SD 3.0–4.9 degrees). The mean side-to-side difference in external rotation between ankles ranged from 1.4 to 3.4 degrees for the 4 observers (range 0.0–12.4 degrees). The mean ratio in external rotation between ankles ranged from 1.1 to 1.5 for the 4 observers (range 0.1–12.1). The mean fibular length ranged from 24.6 to 25.8 mm between the 4 observers (range 13.0–33.6 mm). The mean side-to-side difference in fibular length ranged from 0.5 to 2.1 mm between the 4 observers (range 0.0–12.4 mm). The mean ratio in fibular length between ankles ranged from 1.0 to 1.1 for the 4 observers (range 0.8–2.0).

**TABLE 1.** Mean External Rotation, Difference, and Ratio. And Fibular Length, Difference and Ratio for Different Observers

	Student, n = 166		Resident, n = 166		Surgeon A, n = 66		Surgeon B, n = 66	
	Mean (SD)	Range (min–max)	Mean (SD)	Range (min–max)	Mean (SD)	Range (min–max)	Mean (SD)	Range (min–max)
External rotation, (degrees)	7.7 (4.9)	0.1–22.0	6.7 (4.2)	0.1–19.4	7.7 (3.0)	1.0–15.3	6.6 (3.8)	0.6–18.0
Difference external rotation (degrees)	3.4 (3.1)	0.0–19.7	3.1 (2.4)	0.1–10.7	1.4 (1.4)	0–6.5	1.5 (2.0)	0.0–8.5
Ratio external rotation	1.3 (1.5)	0.1–12.1	1.5 (1.6)	0.0.–10.5	1.2 (1.2)	0.4–7.5	1.1 (0.5)	0.5–3.2
Fibular length (mm)	25.8 (3.1)	16.8–33.6	25.4 (2.7)	17.4–32.1	25.8 (2.6)	20.0–31.6	24.6 (3.1)	13.0–31.0
Difference fibular length (mm)	2.1 (1.7)	0.0–7.0	1.5 (1.3)	0.0–5.7	0.5 (0.6)	0.0–3.0	1.0 (2.0)	0.0–12.4
Ratio fibular length	1.0 (0.1)	0.8–1.4	1.0 (0.1)	0.8–1.2	1.0 (0.0)	0.9–1.1	1.0 (0.2)	0.9–2.0

Mean values of the measurements of 166 ankles (83 patients) by student and resident and 66 ankles (33 patients) by both surgeons.

max, maximum; min, minimum.

### Interobserver Variability

Table 2 summarizes the ICC for interobserver variability. The overall interobserver variability for external rotation was 0.44 (95% CI, 0.32–0.57) and ranged from 0.27 to 0.65 between different observers. The highest ICC for external rotation (0.65) was between the 2 trauma surgeons and the ICCs between the 2 surgeons and the resident were 0.54 and 0.51. The overall interobserver variability for fibular length was 0.59 (95% CI, 0.47–0.70) and ranged from 0.45 to 0.73 between different observers. The highest ICC for fibular length (0.73) was between one of the surgeons and the resident, followed by the ICC between the 2 surgeons (0.60).

**TABLE 2.** Interobserver Reliability Between Different Observers

	ICC All Observers (95% CI)	ICC Surgeon A—Surgeon B (95% CI)	ICC Surgeon A—Resident (95% CI)	ICC Surgeon B—Resident (95% CI)	ICC Surgeon a—Student (95% CI)	ICC Surgeon B—Student (95% CI)	ICC Student—Resident (95% CI)
External rotation	0.44 (0.32–0.57)	0.65 (0.49–0.77)	0.51 (0.31–0.67)	0.54 (0.34–0.69)	0.27 (0.03–0.48)	0.33 (0.10–0.53)	0.51 (0.39–0.61)
Fibular length	0.59 (0.47–0.70)	0.60 (0.42–0.74)	0.73 (0.59–0.82)	0.49 (0.28–0.65)	0.61 (0.44–0.74)	0.45 (0.24–0.62)	0.65 (0.56–0.73)

## DISCUSSION

With a mean of 1.4–3.4 degrees, the differences in fibular rotation between both sides were within a clinically relevant cutoff value of 5 degrees.^32^ The maximum difference between different observers was 1.1 degree. This suggests that this method is reliable and repeatable. This is in concordance with a study from Nault, in which this method was primarily described, although they did not use bilateral CT scans. The importance of bilateral CT scans is shown by the range in both our study and the study conducted by Nault et al.^21^

The large range but relatively small difference between ankles in the same patient shows the validity and importance of using the healthy ankle as a comparison, compared with using a standard value for every patient. Nault et al measured a mean rotation of 6.9 degrees, with a difference of 2.4 degrees between 2 observers. This study measured comparable mean rotations while the mean had a smaller range between observers (mean of 6.6–7.7 degrees). Following the first study by Nault, the Nault talar dome method has been repeated by Schon et al.^15^ This study was conducted in a smaller group, consisting of 12 pairs of ankles, and cadaveric specimens were used instead of living specimens. In 2021, Vetter et al^23^ concluded that the optimal location to measure fibular rotation is between 4 and 6 mm distal to the talar joint line, which is in line with the level of our measurements. The study by Vetter measured the fibular rotation in unilateral CT scans, making the results of this study even more valuable. Recently, Beisemann et al^25^ compared the fibular rotation of a cadaveric specimen with the rotation after an artificially created instability and rotation and reported a similar angle to that reported by Nault et al. Their conclusion was that rotational deviations of ≥10 degrees can be measured using this method when compared with the contralateral uninjured side; however, this remains unsupported since. Furthermore, the comparison with the contralateral side was conducted on fresh frozen legs instead of in vitro measurements with fixed angles using a jig. As it stands, our comparison of bilateral CT scans with low difference between both sides is important for the interpretation of previous studies and is useful to interpret results in a clinical setting.

In addition, this study measured fibular length in both uninjured ankles. Between ankles, the difference in length had a ratio close to 1.0. Furthermore, the maximal mean differences between ankles was 2.1 mm for different observers, although the highest difference was found in the measurements of the least experienced observer.

The range of fibular lengths was relatively large because the shortest fibula was measured at 13.0 mm and the longest at 33.6 mm. This is very likely caused by demographical factors, such as age, gender, or race. Compared with the results observed in the study conducted by Panchbhavi et al, the mean fibular length observed in our study is higher.^24^ This could be due to particulars of our Dutch population, where the height of 19-year-old men and women averaged 182.9 cm and 169.3 cm, respectively (data from 2020).^33^ In comparison, American men average 175.0 cm and American women 161.3 cm in height.^34^ In addition, the difference could be because of our use of CT scan data, compared with the use of plain radiographic imaging by Panchbhavi et al.

Because the difference between ankles within the same patients were only 1.4–3.4 degrees in rotation and 0.5–2.1 mm, this measurement may be useful to detect malposition after fibular fracture fixation with and without syndesmotic injury. Whether or not this fibula tip length is more useful or more accurate than the talocrural or bimalleolar angle would be interesting for future research.^29^

Although the differences in mean rotation were small, the interobserver reliability for the external rotation was poor to moderate. This was because of individual measurements, and these had a larger difference than the mean values. The ICC was higher when measured between more experienced observers compared with the measurements of the student. This indicates that this measurement requires a certain level of skill and should therefore preferably be performed by experienced clinicians.

For the fibular length, the interobserver reliability was moderate to good. This was higher than for the measurements of the external rotation, indicating better interobserver variability. Again, there is an increase in interobserver variability when measured between observers with more experience, although differences are smaller compared with the measurements for external rotation.

Criteria for reconstruction of malunited or malreduced ankles are a shortened fibula of more than 2.0–2.5 mm or rotation difference of more than 5 degrees because these abnormalities are likely to cause a significant change in joint loading and subsequent osteoarthritis in the future.^35–37^ These cutoff values are significantly higher than the interleg difference found in this study, especially the values found by the more experienced observers. This indicated that even smaller abnormalities can be detected with sufficient accuracy, in any case well below the 10 degrees cutoff of that reported in the study conducted by Beisemann et al^25^ Not all surgeons may have a CT scan readily available for postoperative imaging. Therefore, multiple methods have been described to obtain correct per-operative alignment.^38,39^ The bilateral CT scan to measure differences between both ankles is especially useful in cases with uncertainty about (mal)reduction or secondary dislocation, for example, a valgus ankle with a widening of the medial clear spaces.^4^

A limitation of this study is that it is unclear what the effect of foot and ankle positioning on rotation is during the scan. It is well known that the fibula externally rotates approximately 2–3 degrees during dorsiflexion.^40^ One can assume that both legs were scanned in a comparable and neutral position, but some difference in position between left and right leg may account for a small difference in rotation. In future studies, scanning protocols with a jig aiming to scan both ankles in a similar position should account for this effect because both rotational and fibular length measurements are mainly used postoperatively. A second limitation might be that routine postoperative CT scans would expose patients to harmful radiation and that frequent use of the CT scan should therefore be prevented. This would be the case in CT scans of central parts of the body, while a CT scan of the foot or ankle has effective doses of 0.07 microsievert, compared with 19.15 microsievert for spinal CT scans and 0.1 microsievert for a round-trip flight from London to New York.^41,42^

For future perspective, it is not clear what the minimal clinical important difference is in degrees of fibular rotation and millimeters of fibular length. Therefore, by using patient-reported outcome measures and bilateral CT scans after ankle fracture surgery, it may be possible to identify the difference in length and rotation that affects outcome.

## CONCLUSIONS

Using bilateral ankle CT scans, the mean side-to-side difference in fibular rotation using the Nault talar dome method was 1.4–3.4 degrees for different observers. The distal fibular length had a mean side-to-side difference of 0.5–2.1 mm. Although the ICCs were low, the interleg differences in patients were small, making them useful for clinical practice.
